# 2-Methyl-3-(4-methyl­phen­yl)-5,6-diphenyl-2,3-dihydro­pyrazine

**DOI:** 10.1107/S1600536812041438

**Published:** 2012-10-06

**Authors:** A. Thiruvalluvar, N. Anuradha, S. Chitra, D. Devanathan, R. J. Butcher

**Affiliations:** a Postgraduate Research Department of Physics, Rajah Serfoji Government College (Autonomous), Thanjavur 613 005, Tamilnadu, India; b Department of Chemistry, KSR College of Engineering, KSR Kalvi Nagar, Tiruchengode 637 215, Tamilnadu, India; c Department of Chemistry, Government Arts College, C. Mutlur 608 102, Chidambaram, Tamilnadu, India; d Department of Chemistry, Howard University, 525 College Street NW, Washington, DC 20059, USA

## Abstract

In the title mol­ecule, C_24_H_22_N_2_, four atoms (N—C—C—N) of the heterocyclic ring, with their attached H atoms, and all atoms of the methyl group, are disordered over two positions; the site-occupancy factor of the major component is 0.713 (6). The major disorder component of the heterocyclic ring adopts a half-chair conformation, with all substituents equatorial. The benzene ring adjacent to the methyl group forms dihedral angles of 79.68 (11) and 80.92 (11)° with the phenyl rings; the dihedral angle between adjacent phenyl rings is 59.10 (11)°. The crystal structure features three C—H⋯π inter­actions.

## Related literature

For the biological properties of dihydro­pyrazines and for closely related crystal structures, see: Anuradha *et al.* (2009 ▶, 2011 ▶).
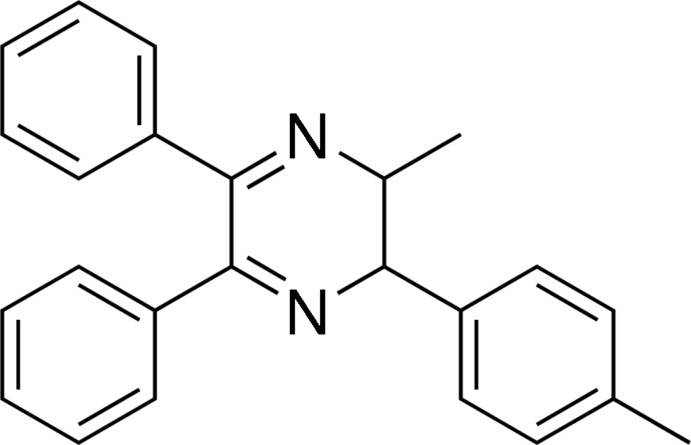


## Experimental

### 

#### Crystal data

C_24_H_22_N_2_
*M* *_r_* = 338.44
Monoclinic, 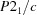

*a* = 8.1986 (5) Å
*b* = 11.8211 (6) Å
*c* = 19.6686 (7) Å
β = 93.638 (4)°
*V* = 1902.38 (17) Å^3^
*Z* = 4
Cu *K*α radiation
μ = 0.53 mm^−1^
*T* = 123 K
0.39 × 0.21 × 0.17 mm

#### Data collection

Agilent Xcalibur Ruby Gemini diffractometer
Absorption correction: multi-scan (*CrysAlis PRO*; Agilent, 2012 ▶) *T*_min_ = 0.830, *T*_max_ = 1.000
12585 measured reflections
3892 independent reflections
3044 reflections with *I* > 2σ(*I*)
*R*_int_ = 0.028

#### Refinement

*R*[*F* ^2^ > 2σ(*F* ^2^)] = 0.069
*wR*(*F* ^2^) = 0.214
*S* = 1.07
3892 reflections
254 parameters
68 restraints
H-atom parameters constrained
Δρ_max_ = 0.71 e Å^−3^
Δρ_min_ = −0.35 e Å^−3^

### 

Data collection: *CrysAlis PRO* (Agilent, 2012 ▶); cell refinement: *CrysAlis PRO*; data reduction: *CrysAlis PRO*; program(s) used to solve structure: *SHELXS97* (Sheldrick, 2008 ▶); program(s) used to refine structure: *SHELXL97* (Sheldrick, 2008 ▶); molecular graphics: *ORTEP-3* (Farrugia, 1997 ▶); software used to prepare material for publication: *PLATON* (Spek, 2009 ▶).

## Supplementary Material

Crystal structure: contains datablock(s) global, I. DOI: 10.1107/S1600536812041438/hg5255sup1.cif

Structure factors: contains datablock(s) I. DOI: 10.1107/S1600536812041438/hg5255Isup2.hkl

Supplementary material file. DOI: 10.1107/S1600536812041438/hg5255Isup3.cdx

Supplementary material file. DOI: 10.1107/S1600536812041438/hg5255Isup4.cml

Additional supplementary materials:  crystallographic information; 3D view; checkCIF report

## Figures and Tables

**Table 1:** Hydrogen-bond geometry (Å, °) *Cg*3 and *Cg*4 are the centroids of the C6–C11 and C12–C17 phenyl rings, respectively.

*D*—H⋯*A*	*D*—H	H⋯*A*	*D*⋯*A*	*D*—H⋯*A*
C10—H10*A*⋯*Cg*4^i^	0.95	2.95	3.743 (3)	142
C19—H19*A*⋯*Cg*4^ii^	0.95	2.77	3.578 (3)	143
C22—H22*A*⋯*Cg*3^iii^	0.95	2.66	3.590 (3)	165

Symmetry codes: (i) 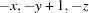; (ii) 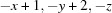; (iii) 
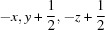
.

**Fig. 1.**
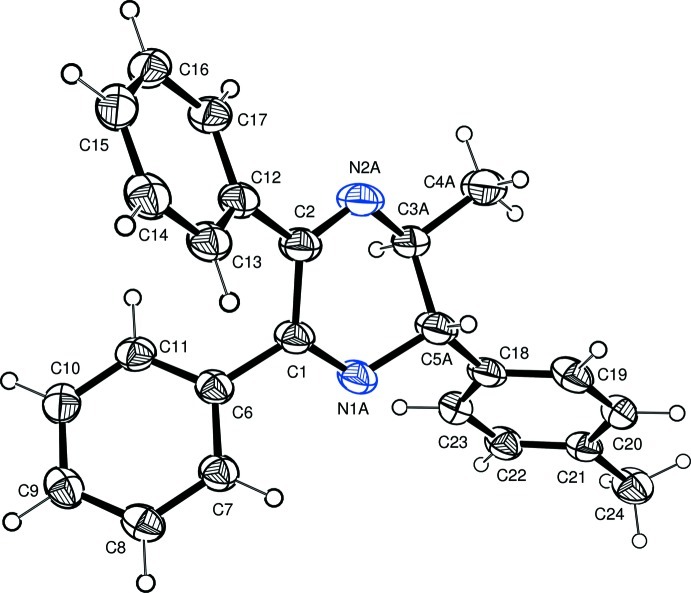
The molecular structure of the title compound, with displacement ellipsoids drawn at the 30% probability level. H atoms are shown as small spheres of arbitrary radius. Only the major disorder component is shown.

## supplementary crystallographic information

### Comment

As part of our investigations of dihydropyrazine derivatives (Anuradha *et al.*, 2009, 2011) to compare their chemical and biological activities, we have undertaken the X-ray crystal structure analysis of the title compound.

In the title molecule, C_24_H_22_N_2_, Fig.1., the heterocyclic ring with the major disorder component, adopts a half-chair conformation, with all substituents equatorial. The phenyl ring at C1 makes a dihedral angle of 59.10 (11) and 79.68 (11)° with the phenyl ring at C2 and benzene ring at C5A respectively. The dihedral angle between the phenyl ring at C2 and benzene ring at C5A is 80.92 (11)°. Four atoms (N2—C3—C5—N1) of the heterocyclic ring, with their attached H atoms, and all atoms of the methyl group, are disordered over two positions; the site occupancy factors refined to 0.713 (6) and 0.287 (6). The crystal structure is stabilized by three C—H···π interactions (Table 1). No hydrogen bonds are found in the crystal structure.

### Experimental

To a homogeneous solution of benzil (1.05 g, 0.005 mol) and 1-methyl-2-(4'-methylphenyl)-ethanediaminedihydrochloride (1.29 g, 0.005 mol) in ethanol (20 ml), sodium acetate trihydrate (2.04 g, 0.015 mol) was added. The precipitated sodium chloride was filtered off and the filtrate was refluxed for 2 h. On completion of the reaction, as indicated by TLC, the reaction mixture was poured into crushed ice and the resulting solid was filtered and purified by column chromatography on silica gel. Elution with benzene-petroleum ether (4:1 *v*/*v*) at 333–353 K gave the pure product. Yield 1.60 g (72%). The pure product was recrystallized in ethyl acetate, to obtain crystals suitable for X-ray diffraction studies.

### Refinement

The H atoms were positioned geometrically and allowed to ride on their parent atoms, with C—H = 0.95–1.00 Å, and with *U*_iso_(H) = 1.2–1.5Ueq(C). Four atoms (N2/C3/C5/N1) of the heterocyclic ring, with their attached H atoms, and all atoms of the methyl group, are disordered over two positions. The anisotropic displacement parameters of equivalent atoms were constrained to be equal; the site occupancy factors refined to 0.713 (6) and 0.287 (6). A damping factor (damp 100 in the final refinement cycles) was applied to avoid large displacements of the disordered hydrogen atoms.

### Crystal data

**Table:**

C_24_H_22_N_2_	*F*(000) = 720
*M**_r_* = 338.44	*D*_x_ = 1.182 Mg m^−^^3^
Monoclinic, *P*2_1_/*c*	Melting point: 450 K
Hall symbol: -P 2ybc	Cu *K*α radiation, λ = 1.54184 Å
*a* = 8.1986 (5) Å	Cell parameters from 3179 reflections
*b* = 11.8211 (6) Å	θ = 3.7–75.3°
*c* = 19.6686 (7) Å	µ = 0.53 mm^−^^1^
β = 93.638 (4)°	*T* = 123 K
*V* = 1902.38 (17) Å^3^	Prism, colourless
*Z* = 4	0.39 × 0.21 × 0.17 mm

### Data collection

**Table:**

Agilent Xcalibur Ruby Gemini diffractometer	3892 independent reflections
Radiation source: Enhance (Cu) X-ray Source	3044 reflections with *I* > 2σ(*I*)
Graphite monochromator	*R*_int_ = 0.028
Detector resolution: 10.5081 pixels mm^-1^	θ_max_ = 75.5°, θ_min_ = 4.4°
ω scans	*h* = −10→9
Absorption correction: multi-scan (*CrysAlis PRO*; Agilent, 2012)	*k* = −13→14
*T*_min_ = 0.830, *T*_max_ = 1.000	*l* = −24→21
12585 measured reflections	

### Refinement

**Table:**

Refinement on *F*^2^	Primary atom site location: structure-invariant direct methods
Least-squares matrix: full	Secondary atom site location: difference Fourier map
*R*[*F*^2^ > 2σ(*F*^2^)] = 0.069	Hydrogen site location: inferred from neighbouring sites
*wR*(*F*^2^) = 0.214	H-atom parameters constrained
*S* = 1.07	*w* = 1/[σ^2^(*F*_o_^2^) + (0.1119*P*)^2^ + 0.6381*P*] where *P* = (*F*_o_^2^ + 2*F*_c_^2^)/3
3892 reflections	(Δ/σ)_max_ = 0.001
254 parameters	Δρ_max_ = 0.71 e Å^−^^3^
68 restraints	Δρ_min_ = −0.35 e Å^−^^3^

### Special details

**Table:**

Geometry. Bond distances, angles *etc*. have been calculated using the rounded fractional coordinates. All su's are estimated from the variances of the (full) variance-covariance matrix. The cell e.s.d.'s are taken into account in the estimation of distances, angles and torsion angles
Refinement. Refinement of *F*^2^ against ALL reflections. The weighted *R*-factor *wR* and goodness of fit *S* are based on *F*^2^, conventional *R*-factors *R* are based on *F*, with *F* set to zero for negative *F*^2^. The threshold expression of *F*^2^ > 2σ(*F*^2^) is used only for calculating *R*-factors(gt) *etc*. and is not relevant to the choice of reflections for refinement. *R*-factors based on *F*^2^ are statistically about twice as large as those based on *F*, and *R*- factors based on ALL data will be even larger.

### Fractional atomic coordinates and isotropic or equivalent isotropic displacement parameters (Å^2^)

**Table:**

	*x*	*y*	*z*	*U*_iso_*/*U*_eq_	Occ. (<1)
N1A	0.2620 (9)	0.8818 (4)	0.1306 (3)	0.0675 (11)	0.713 (6)
N2A	0.2421 (13)	0.9429 (5)	−0.0086 (3)	0.0781 (11)	0.713 (6)
C1	0.2458 (3)	0.80641 (19)	0.08145 (10)	0.0556 (7)	
C2	0.2522 (3)	0.8383 (2)	0.00937 (11)	0.0604 (7)	
C3A	0.2008 (6)	1.0239 (3)	0.04467 (17)	0.0605 (12)	0.713 (6)
C4A	0.2286 (11)	1.1422 (5)	0.0181 (4)	0.0792 (15)	0.713 (6)
C5A	0.3136 (7)	0.9952 (4)	0.1078 (2)	0.0682 (15)	0.713 (6)
C6	0.2095 (3)	0.68882 (18)	0.10250 (10)	0.0518 (6)	
C7	0.2969 (3)	0.64263 (19)	0.15892 (11)	0.0548 (6)	
C8	0.2641 (3)	0.53339 (19)	0.18009 (12)	0.0584 (7)	
C9	0.1433 (3)	0.47028 (19)	0.14630 (11)	0.0590 (7)	
C10	0.0534 (3)	0.5165 (2)	0.09150 (11)	0.0590 (7)	
C11	0.0877 (3)	0.62512 (19)	0.06909 (10)	0.0553 (7)	
C12	0.2806 (3)	0.75362 (19)	−0.04483 (11)	0.0566 (7)	
C13	0.3907 (3)	0.6650 (2)	−0.03313 (11)	0.0601 (7)	
C14	0.4185 (3)	0.5879 (2)	−0.08442 (13)	0.0666 (8)	
C15	0.3345 (3)	0.5981 (2)	−0.14751 (12)	0.0664 (8)	
C16	0.2262 (3)	0.6859 (2)	−0.16006 (12)	0.0649 (8)	
C17	0.1998 (3)	0.7640 (2)	−0.10907 (11)	0.0617 (7)	
C18	0.2782 (4)	1.0771 (2)	0.16632 (11)	0.0667 (8)	
C19	0.3912 (3)	1.16373 (19)	0.17894 (12)	0.0636 (8)	
C20	0.3719 (3)	1.24072 (18)	0.23056 (11)	0.0562 (7)	
C21	0.2402 (3)	1.23338 (19)	0.27160 (10)	0.0548 (7)	
C22	0.1289 (3)	1.1477 (2)	0.25858 (12)	0.0644 (8)	
C23	0.1480 (3)	1.0702 (2)	0.20701 (12)	0.0685 (8)	
C24	0.2223 (4)	1.3153 (2)	0.32921 (13)	0.0775 (10)	
N1B	0.291 (3)	0.8782 (12)	0.1267 (9)	0.0675 (11)	0.287 (6)
N2B	0.220 (4)	0.9433 (14)	−0.0092 (8)	0.0781 (11)	0.287 (6)
C3B	0.2792 (15)	1.0317 (8)	0.0396 (4)	0.0605 (12)	0.287 (6)
C4B	0.195 (3)	1.1406 (13)	0.0178 (12)	0.0792 (15)	0.287 (6)
C5B	0.2419 (19)	0.9953 (10)	0.1111 (5)	0.0682 (15)	0.287 (6)
H7A	0.37917	0.68605	0.18298	0.0657*	
H8A	0.32533	0.50200	0.21814	0.0701*	
H1A	0.08386	1.01444	0.05527	0.0724*	0.713 (6)
H2A	0.15954	1.15427	−0.02381	0.1186*	0.713 (6)
H3A	0.20059	1.19772	0.05242	0.1186*	0.713 (6)
H4A	0.34371	1.15099	0.00839	0.1186*	0.713 (6)
H5A	0.43148	0.99642	0.09751	0.0819*	0.713 (6)
H15A	0.35169	0.54430	−0.18224	0.0797*	
H16A	0.16954	0.69305	−0.20351	0.0778*	
H17A	0.12614	0.82504	−0.11808	0.0740*	
H19A	0.48250	1.16990	0.15174	0.0763*	
H20A	0.44973	1.29963	0.23819	0.0675*	
H22A	0.03708	1.14176	0.28552	0.0772*	
H23A	0.07014	1.01130	0.19956	0.0822*	
H24A	0.10614	1.32498	0.33688	0.1160*	
H24B	0.27968	1.28581	0.37071	0.1160*	
H24C	0.26928	1.38848	0.31757	0.1160*	
H9A	0.12204	0.39527	0.16064	0.0708*	
H10A	−0.03236	0.47404	0.06897	0.0708*	
H11A	0.02715	0.65567	0.03060	0.0663*	
H13A	0.44729	0.65724	0.01028	0.0720*	
H14A	0.49501	0.52833	−0.07626	0.0798*	
H6B	0.39999	1.04078	0.03728	0.0724*	0.287 (6)
H7B	0.12757	1.12787	−0.02437	0.1186*	0.287 (6)
H8B	0.12589	1.16604	0.05365	0.1186*	0.287 (6)
H9B	0.27751	1.19846	0.01009	0.1186*	0.287 (6)
H10B	0.11982	0.99121	0.10842	0.0819*	0.287 (6)

### Atomic displacement parameters (Å^2^)

**Table:**

	*U*^11^	*U*^22^	*U*^33^	*U*^12^	*U*^13^	*U*^23^
N1A	0.109 (3)	0.0531 (11)	0.0414 (12)	−0.0068 (14)	0.0131 (15)	0.0041 (9)
N2A	0.130 (3)	0.0623 (12)	0.0421 (10)	0.0090 (15)	0.0068 (13)	0.0061 (9)
C1	0.0714 (13)	0.0551 (11)	0.0412 (10)	0.0034 (10)	0.0114 (9)	0.0037 (8)
C2	0.0792 (14)	0.0596 (12)	0.0427 (10)	0.0019 (11)	0.0074 (10)	0.0046 (9)
C3A	0.079 (3)	0.0616 (15)	0.0408 (12)	−0.006 (2)	0.0041 (17)	0.0034 (11)
C4A	0.107 (4)	0.0719 (16)	0.0580 (14)	0.006 (2)	0.001 (2)	0.0083 (12)
C5A	0.109 (4)	0.0531 (13)	0.0435 (13)	0.002 (2)	0.012 (2)	0.0063 (10)
C6	0.0628 (12)	0.0521 (11)	0.0420 (10)	0.0023 (9)	0.0151 (8)	0.0009 (8)
C7	0.0601 (11)	0.0573 (12)	0.0477 (10)	−0.0011 (10)	0.0090 (9)	0.0031 (9)
C8	0.0653 (13)	0.0580 (12)	0.0532 (12)	0.0075 (10)	0.0149 (10)	0.0097 (10)
C9	0.0693 (13)	0.0523 (11)	0.0581 (12)	−0.0006 (10)	0.0247 (10)	0.0006 (9)
C10	0.0647 (12)	0.0634 (13)	0.0508 (11)	−0.0094 (10)	0.0192 (10)	−0.0084 (10)
C11	0.0634 (12)	0.0632 (12)	0.0403 (10)	0.0030 (10)	0.0112 (9)	−0.0014 (9)
C12	0.0698 (13)	0.0591 (12)	0.0424 (10)	−0.0054 (10)	0.0150 (9)	0.0026 (9)
C13	0.0699 (13)	0.0636 (13)	0.0483 (11)	−0.0033 (11)	0.0167 (10)	0.0029 (10)
C14	0.0758 (14)	0.0649 (14)	0.0622 (13)	−0.0046 (12)	0.0299 (11)	−0.0002 (11)
C15	0.0770 (14)	0.0694 (14)	0.0561 (13)	−0.0192 (12)	0.0293 (11)	−0.0120 (10)
C16	0.0688 (13)	0.0823 (16)	0.0451 (11)	−0.0186 (12)	0.0166 (10)	−0.0057 (10)
C17	0.0686 (13)	0.0726 (14)	0.0451 (11)	−0.0058 (11)	0.0132 (10)	0.0018 (10)
C18	0.111 (2)	0.0513 (12)	0.0378 (10)	−0.0028 (12)	0.0060 (11)	0.0081 (9)
C19	0.0850 (15)	0.0542 (12)	0.0542 (12)	0.0065 (11)	0.0249 (11)	0.0092 (9)
C20	0.0645 (12)	0.0511 (11)	0.0539 (11)	0.0012 (10)	0.0101 (9)	0.0056 (9)
C21	0.0672 (12)	0.0562 (12)	0.0416 (10)	0.0042 (10)	0.0093 (9)	0.0067 (9)
C22	0.0682 (13)	0.0724 (15)	0.0539 (12)	−0.0063 (12)	0.0141 (10)	0.0115 (11)
C23	0.0898 (17)	0.0626 (13)	0.0522 (12)	−0.0185 (12)	−0.0031 (12)	0.0072 (10)
C24	0.104 (2)	0.0758 (16)	0.0554 (13)	0.0010 (15)	0.0260 (13)	−0.0034 (12)
N1B	0.109 (3)	0.0531 (11)	0.0414 (12)	−0.0068 (14)	0.0131 (15)	0.0041 (9)
N2B	0.130 (3)	0.0623 (12)	0.0421 (10)	0.0090 (15)	0.0068 (13)	0.0061 (9)
C3B	0.079 (3)	0.0616 (15)	0.0408 (12)	−0.006 (2)	0.0041 (17)	0.0034 (11)
C4B	0.107 (4)	0.0719 (16)	0.0580 (14)	0.006 (2)	0.001 (2)	0.0083 (12)
C5B	0.109 (4)	0.0531 (13)	0.0435 (13)	0.002 (2)	0.012 (2)	0.0063 (10)

### Geometric parameters (Å, º)

**Table:**

N1A—C1	1.315 (6)	C20—C21	1.391 (3)
N1A—C5A	1.484 (7)	C21—C22	1.376 (3)
N1B—C5B	1.47 (2)	C21—C24	1.505 (3)
N1B—C1	1.268 (17)	C22—C23	1.383 (3)
N2A—C3A	1.475 (7)	C3A—H1A	1.0000
N2A—C2	1.287 (6)	C3B—H6B	1.0000
N2B—C2	1.316 (17)	C4A—H3A	0.9800
N2B—C3B	1.48 (2)	C4A—H4A	0.9800
C1—C6	1.486 (3)	C4A—H2A	0.9800
C1—C2	1.471 (3)	C4B—H7B	0.9800
C2—C12	1.491 (3)	C4B—H8B	0.9800
C3A—C4A	1.515 (7)	C4B—H9B	0.9800
C3A—C5A	1.538 (6)	C5A—H5A	1.0000
C3B—C4B	1.51 (2)	C5B—H10B	1.0000
C3B—C5B	1.520 (13)	C7—H7A	0.9500
C5A—C18	1.546 (5)	C8—H8A	0.9500
C5B—C18	1.470 (11)	C9—H9A	0.9500
C6—C7	1.394 (3)	C10—H10A	0.9500
C6—C11	1.383 (3)	C11—H11A	0.9500
C7—C8	1.388 (3)	C13—H13A	0.9500
C8—C9	1.377 (3)	C14—H14A	0.9500
C9—C10	1.379 (3)	C15—H15A	0.9500
C10—C11	1.392 (3)	C16—H16A	0.9500
C12—C13	1.392 (3)	C17—H17A	0.9500
C12—C17	1.394 (3)	C19—H19A	0.9500
C13—C14	1.389 (3)	C20—H20A	0.9500
C14—C15	1.386 (3)	C22—H22A	0.9500
C15—C16	1.378 (3)	C23—H23A	0.9500
C16—C17	1.390 (3)	C24—H24B	0.9800
C18—C19	1.393 (4)	C24—H24C	0.9800
C18—C23	1.377 (4)	C24—H24A	0.9800
C19—C20	1.380 (3)		
			
C1—N1A—C5A	114.1 (4)	C5A—C3A—H1A	110.00
C1—N1B—C5B	114.9 (14)	C5B—C3B—H6B	109.00
C2—N2A—C3A	116.3 (5)	N2B—C3B—H6B	109.00
C2—N2B—C3B	115.7 (14)	C4B—C3B—H6B	109.00
N1A—C1—C6	116.2 (3)	C3A—C4A—H3A	110.00
C2—C1—C6	121.89 (19)	H2A—C4A—H4A	109.00
N1B—C1—C6	119.0 (7)	C3A—C4A—H4A	109.00
N1B—C1—C2	118.6 (8)	H2A—C4A—H3A	110.00
N1A—C1—C2	121.8 (3)	C3A—C4A—H2A	109.00
N2A—C2—C1	120.3 (3)	H3A—C4A—H4A	110.00
N2B—C2—C1	119.4 (8)	C3B—C4B—H7B	109.00
N2B—C2—C12	118.2 (7)	C3B—C4B—H9B	109.00
N2A—C2—C12	117.3 (3)	H7B—C4B—H8B	110.00
C1—C2—C12	122.3 (2)	C3B—C4B—H8B	109.00
C4A—C3A—C5A	112.7 (4)	H8B—C4B—H9B	109.00
N2A—C3A—C4A	107.9 (4)	H7B—C4B—H9B	110.00
N2A—C3A—C5A	106.1 (4)	C3A—C5A—H5A	112.00
N2B—C3B—C4B	107.0 (14)	C18—C5A—H5A	112.00
N2B—C3B—C5B	108.9 (11)	N1A—C5A—H5A	112.00
C4B—C3B—C5B	112.9 (12)	C18—C5B—H10B	103.00
C3A—C5A—C18	109.4 (4)	C3B—C5B—H10B	103.00
N1A—C5A—C3A	106.0 (4)	N1B—C5B—H10B	103.00
N1A—C5A—C18	105.7 (4)	C8—C7—H7A	120.00
N1B—C5B—C18	115.2 (10)	C6—C7—H7A	120.00
C3B—C5B—C18	117.0 (9)	C7—C8—H8A	120.00
N1B—C5B—C3B	113.1 (11)	C9—C8—H8A	120.00
C7—C6—C11	119.0 (2)	C10—C9—H9A	120.00
C1—C6—C7	119.1 (2)	C8—C9—H9A	120.00
C1—C6—C11	121.89 (19)	C9—C10—H10A	120.00
C6—C7—C8	120.2 (2)	C11—C10—H10A	120.00
C7—C8—C9	120.4 (2)	C6—C11—H11A	120.00
C8—C9—C10	119.7 (2)	C10—C11—H11A	120.00
C9—C10—C11	120.3 (2)	C12—C13—H13A	120.00
C6—C11—C10	120.4 (2)	C14—C13—H13A	120.00
C2—C12—C13	121.1 (2)	C15—C14—H14A	120.00
C2—C12—C17	120.1 (2)	C13—C14—H14A	120.00
C13—C12—C17	118.8 (2)	C14—C15—H15A	120.00
C12—C13—C14	120.5 (2)	C16—C15—H15A	120.00
C13—C14—C15	119.9 (2)	C17—C16—H16A	120.00
C14—C15—C16	120.3 (2)	C15—C16—H16A	120.00
C15—C16—C17	119.9 (2)	C12—C17—H17A	120.00
C12—C17—C16	120.6 (2)	C16—C17—H17A	120.00
C5A—C18—C19	116.1 (3)	C18—C19—H19A	120.00
C5A—C18—C23	125.8 (3)	C20—C19—H19A	120.00
C19—C18—C23	118.1 (2)	C21—C20—H20A	119.00
C5B—C18—C19	135.8 (6)	C19—C20—H20A	119.00
C5B—C18—C23	105.2 (6)	C21—C22—H22A	119.00
C18—C19—C20	120.7 (2)	C23—C22—H22A	119.00
C19—C20—C21	121.0 (2)	C18—C23—H23A	119.00
C20—C21—C22	117.9 (2)	C22—C23—H23A	120.00
C20—C21—C24	120.9 (2)	H24B—C24—H24C	109.00
C22—C21—C24	121.2 (2)	H24A—C24—H24B	109.00
C21—C22—C23	121.3 (2)	H24A—C24—H24C	109.00
C18—C23—C22	121.0 (2)	C21—C24—H24A	109.00
N2A—C3A—H1A	110.00	C21—C24—H24B	109.00
C4A—C3A—H1A	110.00	C21—C24—H24C	109.00
			
C5A—N1A—C1—C2	−9.5 (7)	C3A—C5A—C18—C23	77.9 (4)
C5A—N1A—C1—C6	174.5 (4)	C1—C6—C7—C8	179.3 (2)
C1—N1A—C5A—C18	164.4 (5)	C11—C6—C7—C8	1.4 (3)
C1—N1A—C5A—C3A	48.4 (7)	C1—C6—C11—C10	−177.9 (2)
C3A—N2A—C2—C1	−8.4 (10)	C7—C6—C11—C10	−0.1 (3)
C2—N2A—C3A—C5A	47.6 (9)	C6—C7—C8—C9	−1.1 (4)
C2—N2A—C3A—C4A	168.7 (7)	C7—C8—C9—C10	−0.6 (4)
C3A—N2A—C2—C12	175.2 (5)	C8—C9—C10—C11	1.9 (4)
C6—C1—C2—C12	−21.6 (4)	C9—C10—C11—C6	−1.6 (3)
N1A—C1—C6—C7	−44.7 (5)	C2—C12—C13—C14	−178.8 (2)
N1A—C1—C2—C12	162.6 (4)	C17—C12—C13—C14	−0.5 (4)
C6—C1—C2—N2A	162.2 (6)	C2—C12—C17—C16	179.6 (2)
N1A—C1—C6—C11	133.2 (4)	C13—C12—C17—C16	1.3 (4)
C2—C1—C6—C11	−42.8 (3)	C12—C13—C14—C15	−0.9 (4)
N1A—C1—C2—N2A	−13.6 (7)	C13—C14—C15—C16	1.5 (4)
C2—C1—C6—C7	139.4 (2)	C14—C15—C16—C17	−0.6 (4)
N2A—C2—C12—C13	137.5 (6)	C15—C16—C17—C12	−0.8 (4)
N2A—C2—C12—C17	−40.8 (6)	C5A—C18—C19—C20	−179.3 (3)
C1—C2—C12—C17	143.0 (2)	C23—C18—C19—C20	−0.5 (4)
C1—C2—C12—C13	−38.8 (4)	C5A—C18—C23—C22	179.4 (3)
N2A—C3A—C5A—N1A	−66.7 (6)	C19—C18—C23—C22	0.6 (4)
C4A—C3A—C5A—C18	61.9 (6)	C18—C19—C20—C21	0.5 (4)
N2A—C3A—C5A—C18	179.8 (4)	C19—C20—C21—C22	−0.7 (3)
C4A—C3A—C5A—N1A	175.4 (5)	C19—C20—C21—C24	178.1 (2)
N1A—C5A—C18—C19	143.0 (4)	C20—C21—C22—C23	0.9 (3)
N1A—C5A—C18—C23	−35.7 (5)	C24—C21—C22—C23	−177.9 (2)
C3A—C5A—C18—C19	−103.3 (4)	C21—C22—C23—C18	−0.9 (4)

### Hydrogen-bond geometry (Å, º)

Cg3 and Cg4 are the centroids of the C6–C11 and C12–C17 phenyl rings, respectively.

**Table:**

*D*—H···*A*	*D*—H	H···*A*	*D*···*A*	*D*—H···*A*
C10—H10*A*···*Cg*4^i^	0.95	2.95	3.743 (3)	142
C19—H19*A*···*Cg*4^ii^	0.95	2.77	3.578 (3)	143
C22—H22*A*···*Cg*3^iii^	0.95	2.66	3.590 (3)	165

Symmetry codes: (i) −*x*, −*y*+1, −*z*; (ii) −*x*+1, −*y*+2, −*z*; (iii) −*x*, *y*+1/2, −*z*+1/2.

## References

[bb1] Agilent (2012). *CrysAlis PRO* Agilent Technologies, Yarnton, England.

[bb2] Anuradha, N., Chitra, S., Thiruvalluvar, A., Pandiarajan, K., Butcher, R. J., Jasinski, J. P. & Golen, J. A. (2011). *Acta Cryst.* E**67**, o2598. 10.1107/S1600536811036336 PMC3201404 22065837

[bb3] Anuradha, N., Thiruvalluvar, A., Pandiarajan, K., Chitra, S. & Butcher, R. J. (2009). *Acta Cryst.* E**65**, o546. 10.1107/S1600536809005042 PMC2968533 21582205

[bb4] Farrugia, L. J. (1997). *J. Appl. Cryst.* **30**, 565.

[bb5] Sheldrick, G. M. (2008). *Acta Cryst.* A**64**, 112–122. 10.1107/S0108767307043930 18156677

[bb6] Spek, A. L. (2009). *Acta Cryst.* D**65**, 148–155. 10.1107/S090744490804362X PMC2631630 19171970

